# Internal Fixation of Osteochondritis Dissecans of the Knee Leads to Good Long-Term Outcomes and High Degree of Healing without Differences between Fixation Devices

**DOI:** 10.3390/jcm8111934

**Published:** 2019-11-10

**Authors:** Simone Perelli, Agustín Rubén Molina Romoli, Matías Costa-Paz, Juan Ignacio Erquicia, Pablo Eduardo Gelber, Juan Carlos Monllau

**Affiliations:** 1 Institut Català de Traumatologia i Medicina de l’Esport (ICATME)—Hospital Universitari Dexeus, Universitat Autònoma de Barcelona, 08028 Barcelona, Spain; juanerquicia@yahoo.com (J.I.E.); pablogelber@gmail.com (P.E.G.); JMonllau@parcdesalutmar.cat (J.C.M.); 2 Research Department, Instituto Universitario del Hospital Italiano de Buenos Aires, Buenos Aires c1181ach, Argentina; agustinmolinaromoli@gmail.com; 3 Department of Knee Arthroscopy, Hospital Italiano de Buenos Aires, Buenos Aires c1181ach, Argentina; matias.costapaz@gmail.com; 4 Department of Orthopaedic Surgery, Hospital de la Sta Creu i Sant Pau, Universitat Autònoma de Barcelona, 08028 Barcelona, Spain; 5 Department of Orthopaedic Surgery, Hospital del Mar, Universitat Autònoma de Barcelona, 08028 Barcelona, Spain

**Keywords:** osteochondritis dissecans, OCD, internal fixation, skeletally mature knee

## Abstract

The aim of the present study is to describe results at long-term follow-up of internal fixation of unstable Osteochondritis Dissecans (OCD) achieved with three different fixation devices in skeletally mature knees. A retrospective cohort study was performed at 5 to 19 years follow-up. Patient-reported questionnaires were collected at the final follow-up. Postoperative X-rays and MRIs were evaluated for healing of the lesion and articular degeneration. An arthroscopic second look was performed in 74.3% of the cases. Failures were reported as reintervention to address the osteochondral lesion or poor functional outcomes at the last follow-up. A total of 39 subjects with a median follow-up of 10.7 years were included. Herbert screws were used in 51.2% of the cases, bioabsorbable nails in 25.7% of the cases and cannulated screws in 23.1% of the cases. No differences were observed in terms of the clinical score (International Knee Documentation Committee (IKDC) *p* = 0.211; Lysholm *p* = 0.197), radiographic union (*p* = 0.102) or radiographic degeneration (*p* = 0.238) between the three different fixation devices. Arthroscopic second look found complete stability of the lesions in all 29 cases evaluated. The mean postoperative Lysholm score was 83 (range = 33–100) and IKDC score was 79 (range = 39–100). Radiographic union was seen in 74% of the cases. Lack of radiographic union was correlated with worst functional scores. A failure rate of 20.5% was found: four reinterventions were performed, and four patients had poor scores at last follow up. This study shows that internal fixation of condylar OCD in skeletally mature patients provides good long-term clinical results and a high degree of healing regardless of the dimensions of the lesion and type of fixation.

## 1. Introduction

Osteochondritis dissecans (OCD) is a common cause of knee pain and dysfunction among skeletally immature and young adult patients. It is characterized by a pathological disruption of the subchondral bone with a secondary repercussion on articular cartilage. The prevalence of OCD has increased over recent years and the latest epidemiological study reported an incidence of 6.1 per 100,000 people/year overall and rising up to 28.0 per 100,000 between 11 and 15 years old. Males have a 2.4-fold peak incidence in comparison to females [1,2]. Despite being a known pathology, the cause is still not well defined. The most accepted hypotheses are a genetic predisposition, a lack of union of secondary ossification centers, inflammatory processes, local ischemia or repeated microtrauma [3]. The latter might be correlated with the increased incidence of this pathology since participation in high-competition sports at very early age has steadily increased.

When the subchondral bone is unable to heal, the focal necrotic area destabilizes the overlying articular cartilage to different degrees going from softening to complete detachment and thereby generating a free intra-articular body in the last stages [3].

Conservative treatment has been shown to be effective in juvenile OCD in patients with open physes and stable osteochondral lesions [4]. However, surgical treatment is indicated in unstable lesions or stable lesions not responsive to conservative treatment [4]. Almost all skeletally mature patients require a surgical treatment given that the loss of vascular supply and interposed fibrous tissue in the subchondral bone lead to a worse prognosis [5]. 

While consistent data on the surgical treatment of OCD in skeletally immature patients exist, few case series about treatment in adult OCD have been published. 

Several techniques have been described to deal with adult OCD, being the internal fixation of the fragment the most used as primary surgery [6]. Few comparisons between different fixation devices have been published.

The purpose of the present study is to describe the long-term follow-up, clinical and radiological results, rate of complications and reoperations after internal fixation of unstable OCD achieved with three different fixation devices in skeletally mature knees. Our hypothesis is that internal fixation of condylar OCD provides, at long-term follow-up, good clinical results and a high percentage of healing regardless of dimensions of the lesions and type of fixation. 

## 2. Method

A retrospective cohort study of patients treated for OCD of the knee with internal fixation was performed in two international teaching centers. A retrospective chart review was executed in the electronic medical records for all patients who had been treated with internal fixation that had a minimum 5 year follow-up. Patients were identified from a list of all knees treated from 1 January 2000 to 1 May 2014. No limits were set on age but distal femoral physes were evaluated to select only skeletally mature subjects. Any kind of trochlear lesion or grade IV lesion not suitable for a fixation was excluded. Unstable grade IV lesions were considered salvageable when at least 3 mm of attached subchondral bone and minimal fragmentation were observed. Patients with an incomplete preoperative MRI or X-ray evaluation were also excluded.

The Clinical Research and Bioethics Committee approved the study, in accordance with the Declaration of Helsinki of 1975 as revised in 2008 (permission n. 202/10).

### 2.1. Data Collection

In line with the protocol of both institutions, a physical examination was carried out prospectively up to 1 year postoperatively. A knee MRI was obtained at the one-year follow-up. X-rays were routinely obtained after the operation, at two months, at six-months and twelve-months follow-ups. The patients were subsequently asked to participate in a telephone interview and to complete patient-reported questionnaires (PRQ). They were the objective International Knee Documentation Committee (IKDC) ligament evaluation form [7] and the Lysholm knee scale [8]. Moreover, the interviews were conducted to investigate clinical failures and to detect any anomalies or inconsistencies in the patients’ responses to the questions, particularly with reference to localized pain, locking or need for further interventions.

For the present study, the patients were also asked to undergo an X-Ray at the last follow up. Information relative to the injury site, the type of surgical approach performed and fixation devices used were compiled. The classification and sizing of the lesions were obtained by means of both preoperative MRI and intraoperative findings in accordance with the International Cartilage Repair Society (ICRS) classification [9]. They are Type I—a stable lesion with a continuous but softened area covered by intact articular cartilage, Type II—a lesion with partial articular cartilage discontinuity and stable when probed. Type III—a lesion with complete articular cartilage discontinuity but with no dislocation (“dead in situ”) and Type IV—an empty defect or a defect with a dislocated fragment or loose fragment within the bed. 

**Figure 1:**
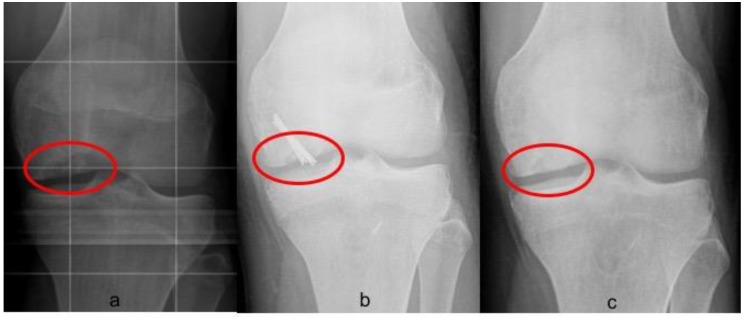
Radiographic union of grade III Osteochondritis dissecans (OCD) of 2.3 cm^2^ in the medial condyle of left knee fixed with two Herbert screws. (**a**) Preoperative evaluation on anteroposterior (AP) view; (**b**) two months postoperative X-ray, not complete union was appreciable; (**c**) six months postoperative X-ray showing a complete union.

**Figure 2:**
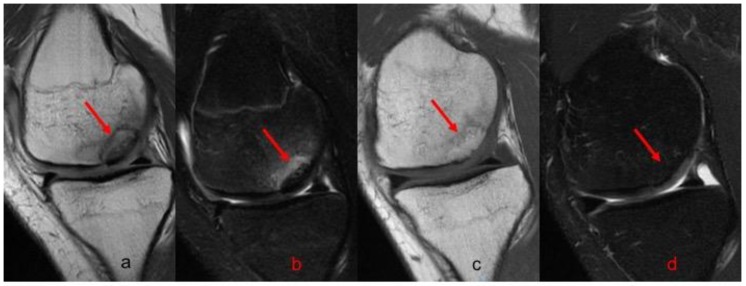
MRI evolution of grade III OCD of 2.9 cm^2^ in the medial condyle of left knee fixed with two Herbert screws. (**a**) T1 preoperative sagittal view; (**b**) T2 preoperative sagittal view; (**c**) Table 1. One-year postoperative sagittal view; (**d**) T2 1 year postoperative sagittal view. In all images the red arrow indicates the lesion.

**Figure 3:**
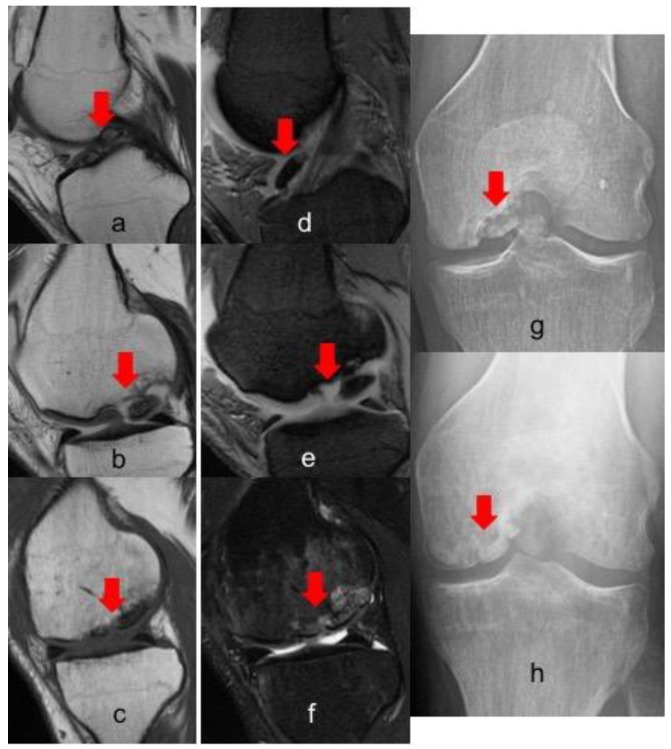
MRI and X-ray evolution of a grade IV OCD of 4.6 cm^2^ in the medial condyle of a right knee fixed with three Smart Nails. (**a**,**b**) T1 preoperative sagittal view; (**d,e**) T2 preoperative sagittal view; (**c**–**f**) 1 year postoperative sagittal view; (**g**) preoperative X-Ray; (**h**) 5 years postoperative X-ray. In all images the red arrow indicates the lesion, in figure a-d the red flag indicates a free intra-articular body.

Postoperative radiographs and MRIs were used to classify and assess the healing of the treated lesion according to previously delineated criteria. (Figure 1, Figure 2 and Figure 3) A fluid interface between the OCD fragment and the condylar bone was considered a sign of incomplete consolidation. Then again, sclerosis or necrosis of the fragment were considered sign of non-consolidation [10,11]. 

Moreover, the articular cartilage status and stability of the lesions were analyzed during implant removal with an arthroscopic second look in 74.3% of the cases.

The radiological evaluation included a weight-bearing anteroposterior (AP) view of the knee in extension, a weightbearing posteroanterior (PA) view at 30° of flexion and a lateral view at 30° of flexion. The joint line was assessed conforming to the Kellgren–Lawrence (KL) classification: Grade 0, no joint space narrowing (JSN) or reactive changes; Grade 1, doubtful JSN, possible osteophytic lipping; Grade 2, definite osteophytes, possible JSN; Grade 3, moderate osteophytes, definite JSN; and Grade 4, large osteophytes, marked JSN [12].

To define a failure, we considered reintervention to address the osteochondral lesion or poor functional outcomes at the last follow-up. The sum of both was generalized as a cumulative failure.

### 2.2. Surgical Technique 

Direct fixation was performed arthroscopically, or it was a mini-open arthrotomy in those cases where the correct approach to the entire lesion was difficult and incomplete only with an arthroscopic view. The procedures were always performed under regional anesthesia and with the use of a tourniquet. Standard anteromedial and anterolateral portals were routinely used. Accessory portals were made when needed according to the site of the lesion to guarantee perpendicular fixation of the osteochondral fragments.

Firstly, a complete evaluation of the knee’s compartments was performed to evaluate the presence of free bodies and associated lesions. The osteochondral lesion was examined to evaluate the integrity of the cartilage surface, the stability of the fragment and the degree of sclerosis of the subchondral bone bed. A probe was used to palpate the lesion to determine its size, stability and consistency of the cartilage surface. In all but two cases, the osteochondral fragment was lifted while keeping the synovial attachment in the notched border of the femoral condyle when possible. The bottom of the lesion was debrided and drilled to obtain a bleeding subchondral surface. In the case of bone defect, a cancellous bone graft from the proximal tibial metaphysis was used to fill it in (six cases). The only two patients on whom an in-situ fixation was carried out had grade 2 lesions, non-responsive to conservative treatment.

**Figure 4:**
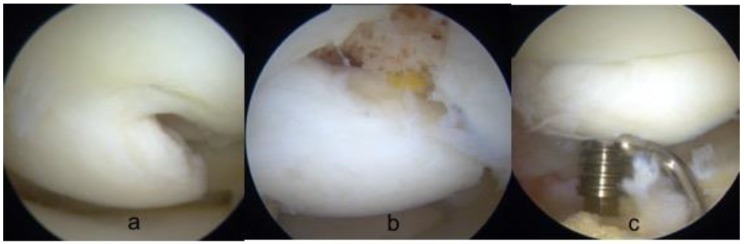
arthroscopic fixation of grade IV OCD. (**a**) The fragment was lifted while keeping a chondral-flap attachment; (**b**) cancellous bone graft was used to fill the bone defect; (**c**) fixation with a cannulated screw was achieved paying attention to direct the screw perpendicular to the lesion.

**Figure 5:**
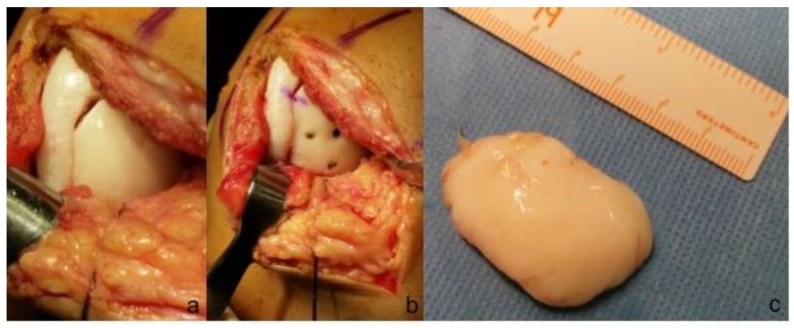
Open reduction and fixation of grade IV OCD with Smart Nails. (**a**) The lesion of the medial condyle was easily individuated; (**b**) after debridement of the subchondral bone the lesion was fixed with four Smart Nails; (**c**) the osteochondral fragment was sized before the fixation.

Once the osteochondral fragment was reduced in its anatomical position with adequate congruence, the fixation was performed. Three different devices where employed: cannulated metal screws (Footmotion, Medcom Tech, Madrid, Spain), Herbert screws (Zimmer-Biomet, Warsaw, USA) or bioabsorbable polylactic nails (Smart Nail, Conmed, Utica, USA). In every case, the aim was to insert the devices perpendicular to the cartilage surface while always avoiding the very central part of the condyle where most of the weightbearing load is. Both the screws head and the nails were always buried below the articular surface in the subchondral bone to gain compression and to avoid protrusion that could erode the opposite tibial surface. The quantity of implants used depended on the size of the osteochondral lesion and on when stability was achieved. (Figure 4 and Figure 5)

Early rehabilitation started from the first postoperative day with continue passive motion without restriction and isometric strengthening of the quadriceps. No weightbearing was allowed during the first 6 weeks. Second look and arthroscopic removal of the screws were performed secondarily. Only in one case was necessary to remove, postoperatively, one absorbable nail because of a secondary breakage of one implant.

### 2.3. Statistical Analysis 

Continuous variables are presented as means, maximums and minimums. Categorical variables are presented as percentages and frequencies. The inference in categorical variables was studied with the chi-square test or Fisher’s exact test depending on what corresponded. The inference in continuous variables was calculated with the paired-samples *T*-test. The level of significance was set at 5% (α = 0.05), bilateral approximation. Correlation analysis was used to explore the associations between the variables. All the analyses were performed with the SPSS 19 (SPSS Inc., Chicago, IL, USA). 

## 3. Results

According to the electronic medical record during the study period, sixty-four patients had undergone OCD treatment with an internal fixation technique. Applying the exclusion criteria, twenty-five patients were excluded. Of those, eight had had repair of trochlear lesions, four patients were unreachable for the telephone interview at last the follow-up, six lacked some of the necessary preoperative images and seven were considered skeletally immature upon evaluating the distal femoral physes. Thus, this retrospective series included thirty-nine subjects, thirty males (76.9%) and nine females (23%). The median age at surgery was 21.6 years (range 14–45). The median follow-up was 10.7 years (range = 5–19). According to the ICRS classification, five of the OCD were grade II (12.8%), twenty-three OCD were grade III (59%) and eleven OCD were grade IV (28.2%), in a stable normally aligned knee. The mean lesion surface area was 3.9 cm^2^ (range = 1.8–8.5). Eleven lesions (28.2%) were located on the lateral femoral condyle, whereas twenty-eight (71.8%) were on the medial condyle. No differences were observed between medial and lateral condyle in terms of the subjective score (IKDC *p* = 0.122, Lysholm *p* = 0.214) or radiographic union (*p* = 0.098). The right knee was involved in eighteen cases (46.1%) and the left knee in twenty-one cases (53.8%). Herbert screws were used for fixation in twenty cases (51.2%), bioabsorbable nails were employed in ten cases (25.7%) and synthesis was carried out using cannulated screws in the remaining nine cases (23.1%). Relevant demographic and surgical characteristics of the study population have been resumed in Table 1. 

No differences were observed in terms of the clinical score (IKDC *p* = 0.211; Lysholm *p* = 0.197), radiographic union (*p* = 0.102) or radiographic degeneration (*p* = 0.238) between the three different fixation devices.

In twenty-nine cases (74.3%), an arthroscopic second look, as well as hardware removal, was carried out at a mean postoperative time of eighty days (range 60–128). Arthroscopic second look evaluation demonstrated complete stability of the osteochondral lesions in 100% of the cases even when radiographic union had not been achieved. Some degree of cartilage fibrillation at the articular surface was detected in 17.8% of the cases without correlation with radiographic union (*p* = 0.238).

In nine cases (23.1%), an open approach was necessary to address the fragment in the proper way. No specific complications were observed in this subgroup of patients and no significative postoperative difference from the arthroscopic group was detected. There was a mean Lysholm (*p* = 0.147) and IKDC (*p* = 0.219) score of 79 and 72, respectively.

At an average follow-up of 10.7 years, the mean overall postoperative Lysholm score was 83 (range = 33–100) and the mean postoperative IKDC score was 79 (range = 39–100).

Radiographic union was seen in 74% of the cases. Both the Lysholm and IKDC score turned out higher in the subgroup with a radiographic union in comparison to the one without radiographic healing (*p* = 0.022; *p* = 0.03). There were no significant differences in healing rate by lesion grade (grade II 100%, grade III 70%, grade IV 73%; *p* = 0.096). Table 2 contains the most important numerical results.

In magnetic resonance images, the fragment was seen properly repositioned in all the knees. Some degree of postoperative MRI alteration was detected in 40.8% of the cases. Signs of incomplete consolidation were detected in 17.9%, the absence of consolidation was present in 7.6% of the patients, subchondral bone edema was present in 5.1% of the cases and cartilage fibrillation in 10.2% of the patients. Patients with any degree of consolidation disturbance in an MRI had inferior IKDC (*p* = 0.028) and Lysholm (*p* = 0.019) scores at the last follow-up. No statistical correlation was observed between any degree of MRI alteration and patient age whereas a weak correlation was observed with the area of the lesions (0.293).

X-rays at five years follow-up were available in 69% of the patients and at ten years follow-up in 53% of the patients. Articular degeneration at the five-year follow-up with the KL classification was: 59.3% grade 0, 35.9% grade 1, 4.8% grade 2. At the ten-year follow-up, it was: 47.4% grade 0, 33.7% grade 1, 13.5% grade 2, 5.4% grade 3. No correlations were seen between the degree of articular degeneration and age at the time of the operation, the dimensions of the lesions or the radiographic union of the osteochondral fragment. 

The most commonly reported complications were hardware backout (one patient, 2.6%), hardware breakage (one patient, 2.6%) and the development of hemarthrosis (nine patients, 23%). In patients where polylactic nails where used, no case of arthro-synovitis was reported, as previously described. The overall cumulative failure rate at 10.7 years follow-up was 20.5% (eight patients), those being four patients who underwent a second intervention and four patients with low functional scores who refused further treatments. In the cases of reintervention, a unicompartmental knee prosthesis was implanted in one patient and three were revised with autologous osteochondral transplantation.

Neither complications or failures were associated specifically with any one of the three devices.

## 4. Discussion

The most important finding of the present study was that internal fixation of condylar OCD in skeletally mature patients provides good long-term clinical results and a high degree of healing regardless of the dimensions of the lesion and type of fixation. Moreover, our results showed a correlation between the radiological union and the long-term results. 

Since it was clear that the simple excision of the osteochondral fragment did not provide good clinical and radiological results [13,14], several options of treatment were proposed.

Moyen et al. reported a failure rate of 53% with isolated screw fixation in adult OCD [15] while this failure rate decreased to 23% in skeletally immature patients with juvenile osteochondritis dissecans. 

Then, some authors proposed adding, in adult OCD, a biological step by performing a hybrid fixation with screws and mosaicplasty at the same time. It showed good long-term functional outcomes and no significant osteoarthritic changes [16]. However, mosaicplasty, both isolated and used in hybrid fixation, is a more invasive surgical procedure and there is an increased cost associated with it. Moreover, with mosaicplasty, there are more technical difficulties in reconstructing the anatomical curvature of the joint surface and almost the entire procedure needs an open approach [17]. Finally, Filardo et al. showed a significant worsening of the Kellgren–Lawrence score of the osteochondral lesion treated with mosaicplasty. This is even more evident when more than one plug is used for the osteochondral reconstruction [18]. So, in dealing with OCD where the mean area of the lesions is around 2.3 and 5.3 cm^2^ [6], mosaicplasty should be considered only as a rescue procedure in case of failure of OCD fixation or in the case of an osteochondral fragment that is not viable for fixation. 

The use of fresh osteochondral allograft would be a perfect solution to provide immediate congruent restoration of the articular surface with structurally competent subchondral bone and the associated viable chondral surface, with no size limitations. That would lead to good clinical results [19]. Unfortunately, the availability of the tissue, the high cost of the technique and some issues around the organization, conservation and distribution of the tissues prevent the spread of this technology.

An alternative surgical procedure for the treatment of osteochondral defects might be biomimetic osteochondral scaffolds or autologous chondrocyte implantation. Both procedures are costly in comparison to internal fixation of the osteochondral fragment in OCD. Moreover, few reports on the long-term results are available on biomimetic scaffolds and conflicting results relative to MRI integration have been published [20,21]. Autologous chondrocyte implantation leads to good long-term results but requires two surgical procedures and additional costs for in vitro cell culture. In addition, data on its use in cases of OCD have shown an incidence of radiographic osteoarthritis (Kellgren and Lawrence score of >2) of 17% at ten years follow-up [22]. 

The results of the present study differ from those of Moyen et al. They showed an overall procedure failure rate of 20.5%, radiographic healing in 74% of the cases, radiographic osteoarthritis KL > 2 in 5.4% of the patients at a mean follow-up of 10.7 years.

Our results match with some other previous results obtained in smaller cohorts of patients. Barrett et al. [23] published the rate of a radiographic union at 82% (18 of 22) after fixation with metal compression screws. Millington et al. [24] reported a healing rate of 67% (12 of 18) after fixation with various bioabsorbable devices. In our series, no difference was observed among the three different fixation devices.

As Barrett et al. [23] described and as seen in our series, there were worse clinical outcomes when the radiographic union was not achieved.

Regarding second-look arthroscopy, both Yonetani et al. [25] and Magnussen et al. [26] reported arthroscopic healing in 100% of the patients (7 and 12, respectively) in their study populations. The results of the present study confirmed that 100% of the fragments revised at second look were stable when probed even when radiographic union had not been achieved.

Although it might be thought that the healing rate in adult OCD is lower than in juvenile OCD, our results confirmed similar outcomes in skeletally mature patients in comparison with the data presented on the skeletally immature.

Kocher et al. [27] studied 24 skeletally immature patients (26 knees) who underwent internal fixation with screws or bioabsorbable devices for unstable grade II–IV lesions. Their study reported radiographic healing in 84.6% (22 of 26 knees), with no significant difference in the healing rate in their patients for the fixation method, lesion location or lesion grade. A recent multicenter study by Wu et al. [28] reported a 76% (66 of 87 patients) overall rate of healing in skeletally immature and mature patients, with no significant difference between cohorts.

Although a similar healing rate has been observed between the adult and skeletally immature populations, only 71.8% of our adult patients had good or excellent results based on their IKDC scoring as opposed to over 90% in studies including skeletally immature patients [29].

### Limitations

The most important limitations of this study are the retrospective design, the small sample size and lack of standardization in terms of the fixation method. The number and configuration of the implants were up to surgeon preference rather than a standard protocol. Moreover, the use of one of the three implants was not randomized but was only due to the variation of the implants over time in our institutions. The small size of the sample made statistical analysis inappropriate for a concrete analysis of the risk factors for failures. 

Finally, the retrospective design leads to a consistent drop out in the long-term X-Ray evaluation, making the interpretation of the data presented about long-term radiographic degeneration doubtful. Nevertheless, this surgery is not common in the adult population, and there are few reports in the literature on the long-term results. Future studies should prospectively evaluate the use of a precise and reproducible fixation technique to confirm or contradict the data presented in this article.

## 5. Conclusions

Internal fixation of condylar OCD in skeletally mature patients provides good long-term clinical results and a high degree of healing regardless of the dimensions of the lesion and type of fixation.

Although good alternative treatments options are available, our data support the use of internal fixation regardless of the fixation device to save the osteochondral fragment. This kind of surgical treatment is technically simple, safe and cheap. Those advantages lead us to think that a cost-effectiveness analysis of its results would show high cost-efficacy. 

## Figures and Tables

**Table 1:** Cross-sectional table demonstrating the relevant characteristics of the study population.

Age at Index Surgery (Years)	Gender	Laterality	ICRS	Lesion Area (cm^2^)	Follow-Up (Years)	Type of Fixation	IKDC	Lysholm	X-Ray Union	Approach
25	Male	Medial	4	2.9	17	2 Herbert Screw	75	75	YES	Arthroscopic
22	Male	Medial	2	3.2	16	2 Herbert Screw	89	90	YES	Arthroscopic
17	Male	Lateral	2	3.1	15	2 Herbert Screw	93	100	YES	Arthroscopic
35	Male	Medial	3	2.8	19	2 Herbert Screw	39	33	NO	Arthroscopic
15	Female	Lateral	3	2.8	9	2 Smart Nail	45	55	NO	Open
21	Male	Lateral	3	4.1	7	3 Smart Nail	80	85	YES	Arthroscopic
15	Male	Medial	3	3.2	8	2 Smart Nail	89	96	YES	Arthroscopic
41	Male	Medial	3	2.2	17	1 Herbert Screw	59	61	NO	Arthroscopic
16	Male	Medial	3	3.4	14	2 Herbert Screw	83	95	NO	Arthroscopic
22	Male	Medial	3	4.3	14	3 Herbert Screw	92	100	YES	Arthroscopic
26	Male	Medial	3	2.9	12	2 Herbert Screw	81	85	NO	Arthroscopic
22	Male	Medial	4	4.6	19	3 Herbert Screw	74	75	YES	Open
32	Female	Medial	2	3	12	2 Herbert Screw	92	100	YES	Arthroscopic
15	Male	Lateral	4	4.1	13	3 Herbert Screw	49	55	NO	Arthroscopic
18	Male	Medial	3	3.1	6	2 Herbert Screw	81	90	YES	Open
23	Male	Medial	3	3.9	13	2 Cannulated Screw	75	80	YES	Arthroscopic
45	Male	Medial	4	4.3	10	3 Helbert Screw	61	72	YES	Arthroscopic
26	Male	Lateral	3	4.6	8	3 Herbert Screw	100	100	YES	Arthroscopic
15	Male	Lateral	3	3.1	6	2 Helbert Screw	80	86	YES	Arthroscopic
18	Male	Lateral	3	4.6	5	3 Cannulated Screw	59	67	NO	Arthroscopic
18	Male	Medial	4	4.4	5	3 Cannulated screw	59	67	YES	Arthroscopic
17	Female	Medial	3	3.2	12	2 Herbert Screw	87	91	YES	Arthroscopic
20	Male	Medial	3	4.6	12	3 Helbert Screw	75	79	NO	Arthroscopic
21	Male	Medial	4	4.5	11	3 Helbert Screw	79	84	YES	Open
18	Male	Lateral	3	1.8	11	1 Herbert Screw	100	100	YES	Arthroscopic
17	Male	Medial	4	2.8	10	2 Cannulated Screw	76	80	YES	Arthroscopic
15	Female	Medial	4	4.7	9	3 Cannulated Screw	90	93	YES	Arthroscopic
19	Male	Lateral	3	3.2	8	2 Smart Nail	86	89	YES	Arthroscopic
22	Male	Medial	3	4	7	3 Smart Nail	85	88	YES	Arthroscopic
30	Female	Lateral	4	5.8	7	4 Smart Nail	63	69	YES	Open
14	Female	Lateral	2	2.3	6	1 Helbert Screw	90	94	YES	Arthroscopic
20	Male	Medial	3	8.4	5	4 Cannulated Screw	68	76	NO	Open
23	Female	Medial	4	4.6	5	3 Smart Nail	79	84	NO	Open
32	Male	Medial	4	4.2	15	2 Cannulated Screw	87	91	YES	Arthroscopic
19	Female	Medial	3	8.5	14	4 Smart Nail	85	91	YES	Open
17	Male	Medial	2	2	14	1 Cannulated Screw	85	89	YES	Arthroscopic
21	Male	Medial	3	3.4	13	2 Cannulated Screw	95	100	YES	Arthroscopic
15	Female	Medial	3	5.9	7	4 Smart Nail	86	88	YES	Open
17	Male	Medial	3	4.9	6	3 Smart Nail	75	79	YES	Arthroscopic

**Table 2:** Numerical results.

	IKDC Score	LHYSOLM Score	X-RAY Complete Union
**Medial VS.** **Lateral Condyle**	Mean 76.6 (SD 2.6) VS. 78.4 (SD 3.2)	Mean 81.8 (SD 3.1) VS. 83.3 (SD 3.3)	75% VS. 72.7%
**Open VS.** **Arthroscopic Surgery**	Mean 72.7 (SD 2.4) VS. 79.4 (SD 4.9)	Mean 79.2 (SD 2.8) VS. 83.8 (SD 5.3)	66.7% VS. 76.5%
**Herbert screw VS. Cannulated screw VS. Absorbable Nails**	Mean 78.8 (SD 3.2) VS. 77.1 (SD 3.6) VS. 77.3 (SD 4.1)	Mean 83.3 (SD 2.8) VS. 82.5 (SD 3.3) VS. 82.4 (SD 3.6)	70% VS. 77.6% VS. 80%
**X-ray Union VS.** **X-ray Non-Union**	Mean 64 (SD 3.9) VS. 82.9 (SD 2.3)	Mean 68.9 (SD 3.5) VS. 87.7 (SD 2.5)	
